# Grouping Annotations on the Subcellular Layered Interactome Demonstrates Enhanced Autophagy Activity in a Recurrent Experimental Autoimmune Uveitis T Cell Line

**DOI:** 10.1371/journal.pone.0104404

**Published:** 2014-08-12

**Authors:** Xiuzhi Jia, Jingbo Li, Dejing Shi, Yu Zhao, Yucui Dong, Huanyu Ju, Jinfeng Yang, Jianhua Sun, Xia Li, Huan Ren

**Affiliations:** 1 Department of Immunology, Harbin Medical University, Harbin, China; 2 Infection and Immunity, Key Laboratory of Heilongjiang Province, Harbin, China; 3 College of Bioinformatics Science and Technology, Harbin Medical University, Harbin, China; 4 Department of Anesthesiology, the 2nd Affiliated Hospital of Harbin Medical University, Harbin, China; 5 Department of Ophthalmology, the 4th Affiliated Hospital of Harbin Medical University, Harbin, China; 6 The Blood Center of Hei Long Jiang Province, Harbin, China; University of Chicago, United States of America

## Abstract

Human uveitis is a type of T cell-mediated autoimmune disease that often shows relapse–remitting courses affecting multiple biological processes. As a cytoplasmic process, autophagy has been seen as an adaptive response to cell death and survival, yet the link between autophagy and T cell-mediated autoimmunity is not certain. In this study, based on the differentially expressed genes (GSE19652) between the recurrent versus monophasic T cell lines, whose adoptive transfer to susceptible animals may result in respective recurrent or monophasic uveitis, we proposed grouping annotations on a subcellular layered interactome framework to analyze the specific bioprocesses that are linked to the recurrence of T cell autoimmunity. That is, the subcellular layered interactome was established by the Cytoscape and Cerebral plugin based on differential expression, global interactome, and subcellular localization information. Then, the layered interactomes were grouping annotated by the ClueGO plugin based on Gene Ontology and Kyoto Encyclopedia of Genes and Genomes databases. The analysis showed that significant bioprocesses with autophagy were orchestrated in the cytoplasmic layered interactome and that mTOR may have a regulatory role in it. Furthermore, by setting up recurrent and monophasic uveitis in Lewis rats, we confirmed by transmission electron microscopy that, in comparison to the monophasic disease, recurrent uveitis *in vivo* showed significantly increased autophagy activity and extended lymphocyte infiltration to the affected retina. In summary, our framework methodology is a useful tool to disclose specific bioprocesses and molecular targets that can be attributed to a certain disease. Our results indicated that targeted inhibition of autophagy pathways may perturb the recurrence of uveitis.

## Introduction

It is estimated that approximately 10% of the blindness cases in the United States have been caused by uveitis[1], [2]. The disease often shows a relapsing–remitting course and is associated with T cell-mediated autoimmune responses; however, the mechanism underlying the relapsing–remitting course of this disease remains undetermined. Autoantigen-induced experimental autoimmune uveitis (EAU) in Lewis rats serves as a model for the clinical heterogeneity of human uveitis. Research data show that different autoantigens may induce different disease phenotypes; for example, the retinal-soluble antigen (S-Ag), interphotoreceptor retinoid-binding protein (IRBP) or their peptide derivative- (PDSAg from S-Ag and R16 from IRBP) specific T cell lines induce a monophasic disease course, whereas R14- (a peptide derived from IRBP) specific T cell lines mediate a typical recurrent disease process when adoptively transferred to a susceptible animal [3]. Although the mechanisms that underlie recurrent human uveitis remain unclear,Regulatory T cells (Tregs) that selectively restrain the PDSAg-specific T cells and protect from recurrent disease have not been identified and the underlying cause for the relapsing phase of the disease appears to originate in large part in subtle differences in the specific effector phenotype of T cells generated [3]. Therefore, the different antigen-specific T cell lines may allow a direct comparison between the recurrent and monophasic phenotypes of the disease to elicit the initiation element to induce the recurrence of uveitis.

Complex cellular activity is coordinately regulated by protein interaction networks. In recent years, technological advances in the area of omics research have been successful in demonstrating biological networks that closely relate to phenotypic changes[4]. These technological advances include the development of high-throughput screening methods, which are used to profile gene expression data to reconstruct biological networks, thus elucidating the underlying molecular mechanisms of relevant cellular activity[5]. Furthermore, because a given protein may have diverse functions not only tightly linked with its subcellular distribution but also with the proteins with which it interacts, information concerning the subcellular localization and interactions among proteins are important for recreating the model networks that may reflect details of the intricate biological processes at the spatial level[6], [7]. Several public databases are available for analysis of protein interactions, including Network of Functional Coupling (FunCoup) and Human Protein Reference Database (HPRD)[8], [9]. In this study, using FunCoup, we constructed an initial interactome on the basis of the supposed proteins that are encoded by differentially expressed genes between the R14- and S-antigen-specific T cell lines. In addition, the software Cytoscape and its plugins, **Cerebral** (Cell Region-Based Rendering and Layout)[10] **and ClueGO** [11], were applied to create a layered **interactome** by distributing the node proteins into different layers of their respective subcellular localizations[12], [13]. Thus, the most significantly emerging bioprocess related to autophagy in the cytoplasm was obtained.

**Figure 1:**
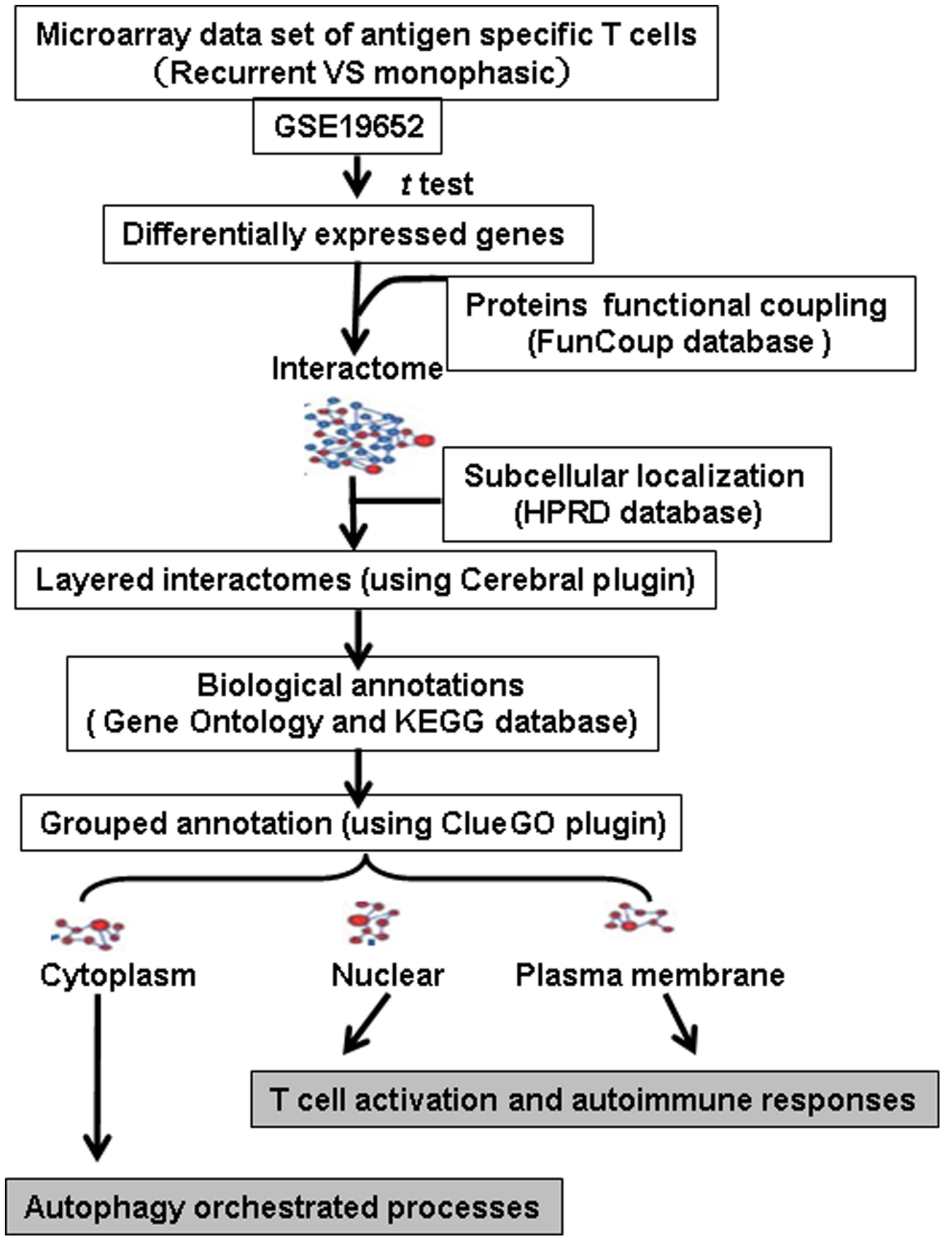
To reveal the specific bioprocess leading to the recurrence of uveitis, we proposed a framework, grouping annotations on the subcellular layered interactome. Annotation gene sets were obtained based on differential expression, subcellular location, and interactome information, and then a functionally organized Gene Ontology/pathway annotations network or module was profiled to reveal autophagy-related biological themes.

Autophagy is an essential homeostatic process in which cells break down their own components through lysosomal machinery[14]. In complex multicellular organisms, the core molecular machinery of autophagy orchestrates diverse aspects of cellular homeostasis and energetic balance in response to dangerous stimuli, such as nutrient deprivation, hypoxia, and infection. A recent study revealed a crucial role of the autophagy pathways and related proteins in immunity and inflammation[15], [16]. Of potential interest to autoimmunity, molecules that are involved in such processes are found to profoundly affect T cell homeostasis[17]. For example, it has been recently revealed that autophagy may promote secretion of IFN-γ and maintenance of homeostasis of both the endoplasmic reticulum and the mitochondria in activated T cells[15]. In this study, we report the framework (demonstrated in Figure 1) through establishing a layered and annotation grouped interactome based on the differential gene expression profiles between the two types of T cell lines that may induce either recurrent or monophasic EAU. Our analyses indicated that the recurrent T cell line showed significantly increased autophagy activity compared to the monophasic line, and these results were also testified *in vivo* by **transmission electron microscopy (TEM)**.

## Results

### Re-organization of the differentially expressed genes by layered interactome

**Figure 2:**
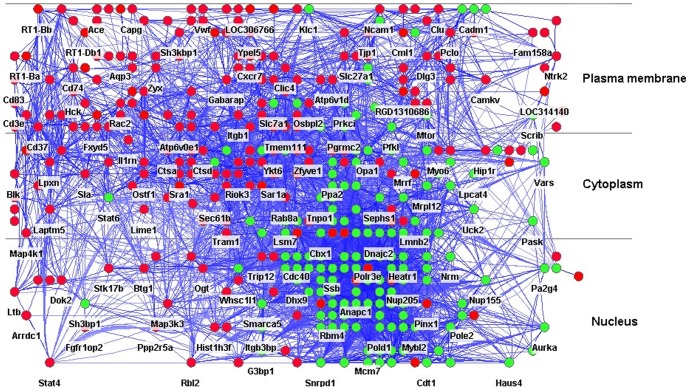
The layered interactome. An initial interactome was assembled using Cytoscape by merging the FunCoup (functional coupling between proteins) database with proteins encoded by the differentially expressed genes between the recurrent and monophasic T cell lines. The protein subcellular localization retrieved from the Human Protein Reference Database was imported as a node attribute. Cytoscape and its plugin Cerebral were applied to redistribute the nodes based on subcellular localization; thus, the interactome was further divided into three layers: the plasma membrane, the cytoplasm and the nucleus. Red and green nodes represent the up-regulated and down-regulated genes.

After a total of 592 differentially expressed genes were obtained when the recurrent T cell line was compared with the monophasic T cell line, the CLICK algorithm was initially used to separate such genes into two clusters: 346 up-regulated genes, 244 down-regulated genes, and two ignored genes that could not be clustered. The 590 genes were then incorporated into the FunCoup database to construct a context-specific interactome in which single node and small components (less than five connected nodes) were removed and only the largest component was left. The adjusted interactome included 339 nodes; of these, 191 were up-regulated and 148 were down-regulated (Table 1). These were further layered using Cytoscape and its Cerebral plugins, which distributed the node proteins into three different layers: the plasma, the cytoplasm, and the nucleus (Figure 2). Information regarding the proteins' subcellular localization was retrieved from the HPRD. The number of proteins encoded by the differentially expressed genes was roughly equivalent among the three different layers (36.6%, 31.6%, and 31.8%, respectively). On further examining the number of proteins in each layer, we found that the sum of the number of proteins encoded by the up-regulated genes was greater than that encoded by the down-regulated genes in the plasma membrane, whereas the opposite trend was observed in the nucleus; the number of up-regulated and down-regulated genes were equivalent in the cytoplasm (Table 1).

**Table 1:** Distribution of nodes in three different layers.

Localization	Up-regulated	Down-regulated	Total%
Plasma membrane	106	18	36.6
Cytoplasm	58	49	31.6
Nucleus	27	81	31.8
Total	191	148	339

### Annotation grouping reveals differential functional modules in the subcellular layers

**Figure 3:**
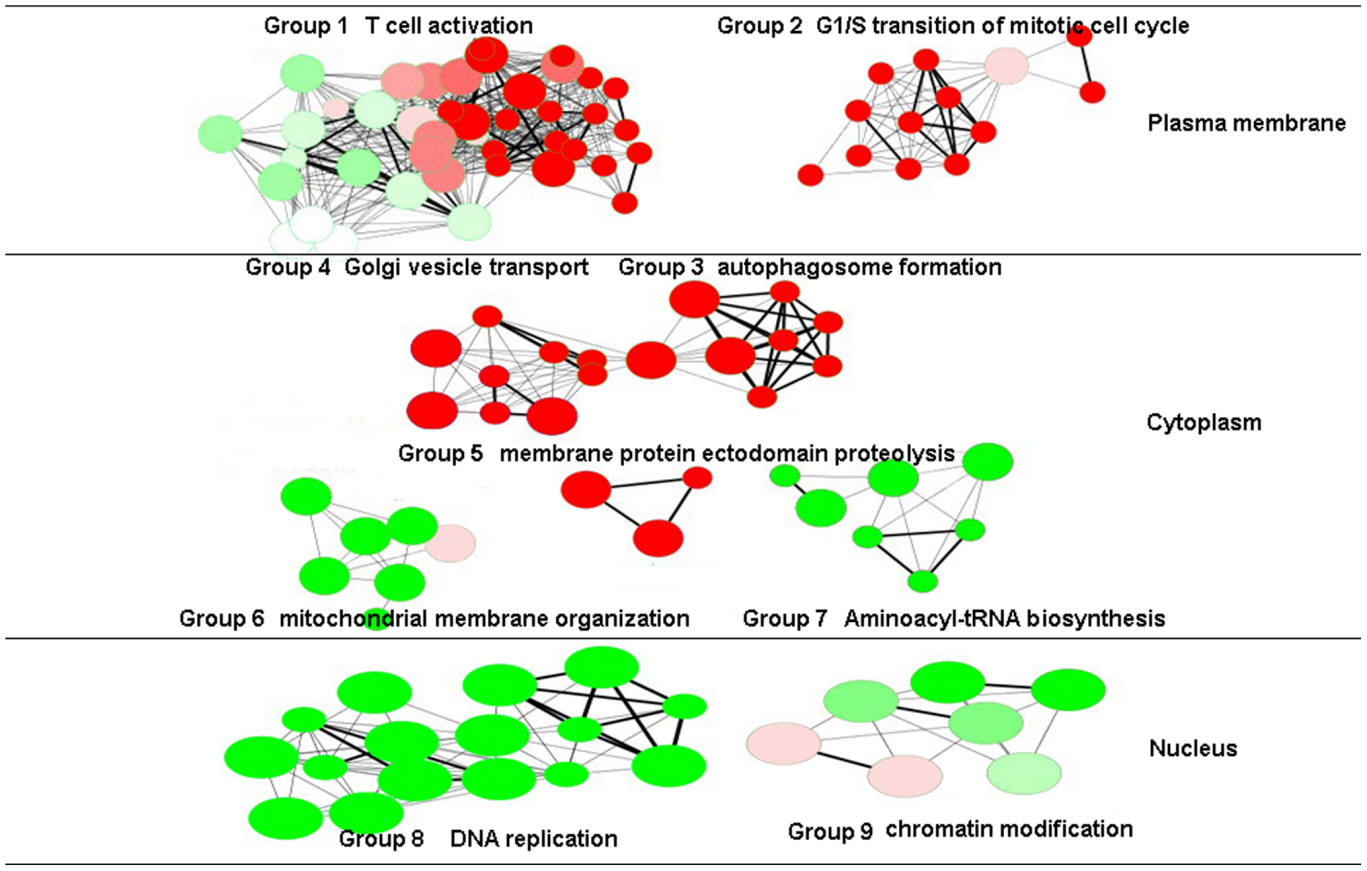
Grouped annotations in layered interactome. The differential genes in different interactomes were annotated in the context of the GO and KEGG databases, and the relationships among these annotated terms were calculated and grouped by ClueGO to create an annotation module network. The created network represented the terms as nodes that were linked based on a predefined kappa score level (>0.3). The size of the nodes reflected the enrichment significance of the terms (from 5×10^−2^ to 5×10^−6^). The most significantly up-regulated (red nodes) and/or down-regulated (green nodes) annotated terms for each group were chosen as the labels of the groups and are shown in bold-face type, while unchanged terms are represented as pink nodes.

To assess the layered interactomes in the context of the Gene Ontology(GO)and Kyoto Encyclopedia of Genes and Genomes (KEGG) databases, the DAVID functional annotation tool was applied to identify significantly over-represented biological processes. In addition, the ClueGO plugin was applied to group these functional annotations and recognize the relationships among these processes (Tables S1–S3 in File S1). Only the most significantly up-regulated or down-regulated terms per group are shown in Table 2. Nine groups of annotations that represented over-expressed biological processes in the three different layers were conclusively identified by ClueGO analysis, including five groups that primarily indicated autophagy-associated processes in the cytoplasm; and four groups of annotations, two in the plasma membrane and two in the nucleus (Figure 3).

**Table 2:** Grouped annotations in layered interactome.

ID	Go Term	Layer	Up/Down Regulated	Bonferroni *P* Value
**Group 1**				
GO:00025040	**Antigen processing and presentation of peptide** **or polysaccharide antigen via MHC class II**	Plasma Membrane	Down Regulated	2.11E-04
GO:00421100	**T cell activation**	Plasma Membrane	Up Regulated	0.005540
**Group 2**				
GO:00000820	**G1/S transition of mitotic cell cycle**	Plasma Membrane	Up Regulated	0.078029
**Group 3**				
GO:00000451	**Autophagosome formation**	Cytoplasm	Up Regulated	0.025024
**Group 4**				
GO:00481931	**Golgi vesicle transport**	Cytoplasm	Up Regulated	0.015571
GO:00068881	*ER to Golgi vesicle-mediated transport*	Cytoplasm	Up Regulated	0.020495
GO:00421471	*Retrograde transport, endosome to Golgi*	Cytoplasm	Up Regulated	0.032053
**Group 5**				
GO:00065091	**Membrane protein ectodomain proteolysis**	Cytoplasm	Up Regulated	0.048607
**Group 6**				
GO:00070061	**Mitochondrial membrane organization**	Cytoplasm	Down Regulated	2.31E-04
GO:00070051	*mitochondrion organization*	Cytoplasm	Down Regulated	0.002982
GO:00650021	**Intracellular protein transmembrane transport**	Cytoplasm	Up Regulated	9.62E-04
**Group 7**				
KEGG:009701	**Aminoacyl-tRNA biosynthesis**	Cytoplasm	Down Regulated	0.024672
**Group 8**				
GO:00062602	**DNA replication**	Nucleus	Down Regulated	0.006732
**Group 9**				
GO:00165682	**Chromatin modification**	Nucleus	Down Regulated	5.63E-06
GO:00064792	**Protein amino acid methylation**	Nucleus	Up Regulated	0.008477

Parallel to the five inter-related annotation groups in the cytoplasm, the groups in the plasma membrane and nucleus indicated significantly active processes that were involved in T cell activation and autoimmune responses. For example, the up-regulated “T cell activation” (group 1, *p* = 0.0055) and “G1/S transition of mitotic cell cycle” (group 2, *p* = 0.0780) in the plasma membrane indicated events that were related to increased T cell receptor activation and T cell proliferation (Table 2). Furthermore, in the nucleus, annotations, such as “chromatin modification” and “protein amino acid methylation” (Table 2, group 9, *p*<0.0085), may characterize epigenetic alterations that may lead to large spatial and temporal changes in gene regulations in autoimmunity[18]–[20]. Taken together, these results demonstrated that, based on the differential gene expression profiles between the recurrent and monophasic T cell line, our integrated analysis identified processes that were significantly representative of autoimmune responses.

### The autophagy-orchestrated processes in the cytoplasm

**Figure 4:**
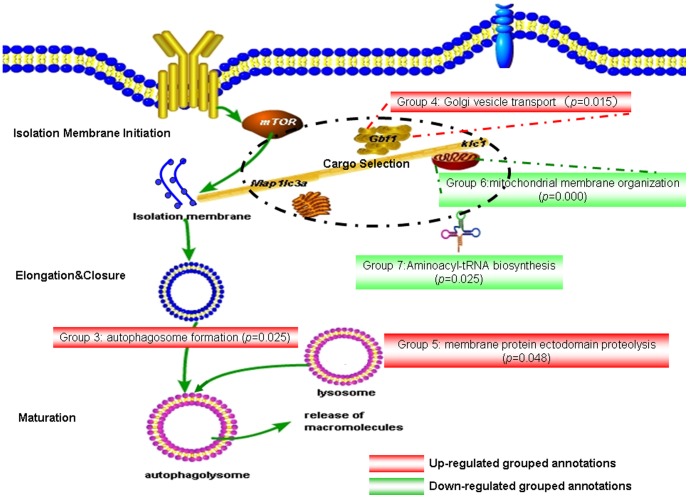
Demonstration of autophagy-orchestrated processes in the cytoplasm. The autophagy process can be broken down into four steps: isolation membrane initiation, elongation and closure, cargo selection, and maturation. The grouped annotations (groups 3–7) analyzed by ClueGO in the cytoplasm layer were shown to be either directly or indirectly associated with autophagy. Annotations of up-regulated genes are shown in red background and those of down-regulated genes are in green background.

**Figure 5:**
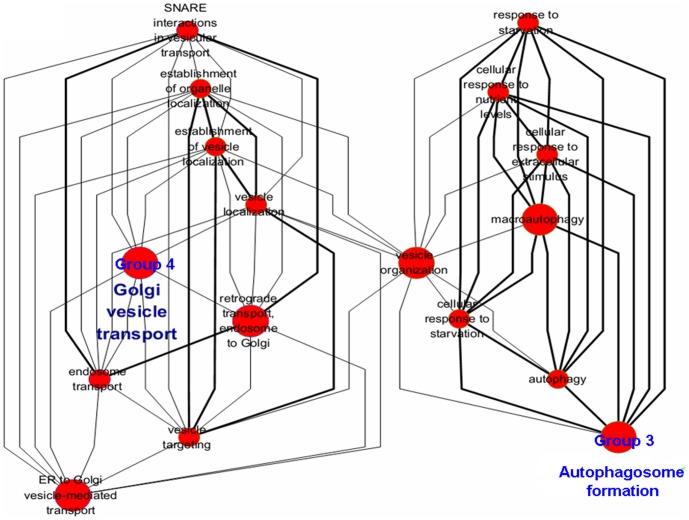
“Golgi vesicle transport” (group 4) and “Autophagosome formation” (group 3) were linked by ClueGO analysis. “Golgi vesicle transport” (group 4, *p* = 0.0156) and “Autophagosome formation” (group 3, *p* = 0.0250) showed close functional correlation in ClueGO analysis and were linked by “vesicle organization.” The nodes represent the annotated terms; the size of the nodes reflects the statistical significance of the term. The degree of connectivity between terms was calculated using kappa statistics, and the threshold was set at 0.3.

Autophagy is a catabolic process that can be divided into four processes: isolation membrane initiation, elongation and closure, cargo selection, and autophagolysosome maturation (Figure 4). Strikingly, all annotations in the five groups in the cytoplasm significantly showed courses that had either direct or indirect linkage with these autophagy-related processes. For example, while the up-regulated “autophagosome formation” (group 3, *p* = 0.0250) was annotated directly, the other four groups of annotations (groups 4–7; Table 2, Figures 3,4) showed obvious connections with increased autophagy activity in the recurrent T cell line. The coordinately up-regulated “Golgi vesicle transport,” “ER to Golgi vesicle-mediated transport,” and “Retrograde transport, endosome to Golgi” (group 4, *p* = 0.0156), and down-regulated “mitochondrial membrane organization” (group 6, *p* = 0.0002) imply that the recurrent T cell line may undergo organelle dissociation, which was reported to initiate isolation of membranes[21]. Consistently, processes of “autophagosome formation” (group 3) and “Golgi vesicle transport” (group 4) that were usually tied by “vesicle organization” were also closely associated with isolation membrane initiation (Figure 5). Furthermore, the down-regulated “mitochondrial membrane organization” and “mitochondrion organization” (group 6, *p* = 0.0002) indicated that the recurrent T cell line may undergo mitochondrial dissociation to accommodate bioenergetic requirements upon TCR engagement[17]. “Aminoacyl-tRNA biosynthesis” (group 7, *p* = 0.0247), a highly energetically favorable process, was also steadily down-regulated. In addition, “membrane protein ectodomain proteolysis” (group 5, *p* = 0.0486) was also a highly autophagy-associated event. Collectively, in the cytoplasm layer, all these annotation-defined processes indicated that the recurrent T cell line may experience an energy shortage state, and autophagy orchestrated multiple organelle activity vigorously.

### Autophagy-annotated gene-related network

**Figure 6:**
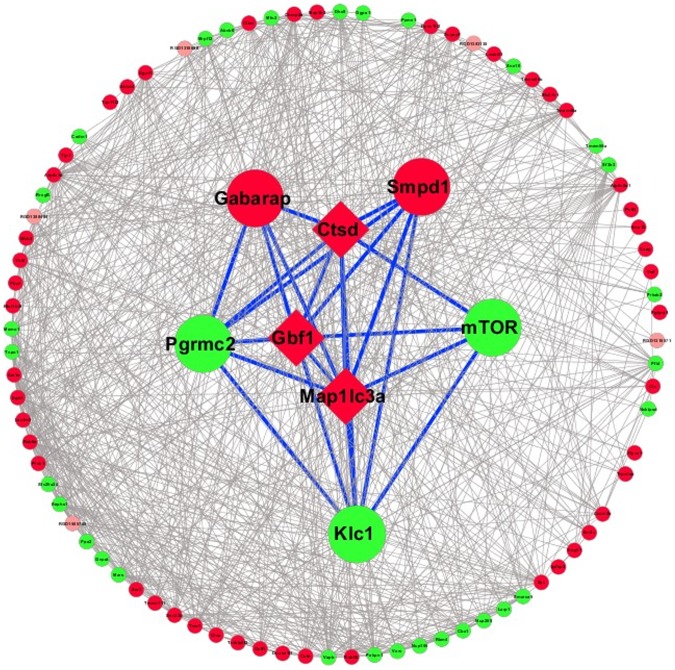
Autophagy-associated networks. Autophagy-associated networks were generated by Cytoscape based on functional coupling of proteins (genes) retrieved from the FunCoup database. Proteins that have connections with autophagy-annotated genes–*Ctsd*, *Gbf1*, and *Map1lc3a* (group 3, Table S2 in File S1)–are shown in the networks. Ctsd, Gbf1, and Map1lc3a are used as the “seeds” and placed at the center of the network surrounded by five proteins that are connected with all these seeds; other proteins with less than three connections to these seeds are shown in a circle. Seeds are depicted as diamond-shaped boxes. Red and green nodes indicate the up-regulated and down-regulated genes, respectively.

Since the annotated grouped interactome was originally obtained from the FunCoup database, which curates detailed functional coupling between proteins, we succeeded in creating a network view of the autophagy-associated processes and exploring the regulatory mechanisms. In this type of analysis, the autophagy-annotated genes–*Ctsd*, *Gbf1*, and *Map1lc3a* (group 3, Table S2 in File S1)–were used as “seeds,” whose functional coupling proteins were extracted from the interactome by FunCoup. Each of the following five proteins–gamma-aminobutyric acid type A receptor-associated protein (*Gabarap*), sphingomyelin phosphodiesterase 1 acid lysosomal (*Smpd1*), mammalian target of rapamycin (*mTOR*), progesterone receptor membrane component 2 (*Pgrmc2*), and kinesin light chain 1 (*Klc1*)–showed a direct functional interaction with all of the three seeds and were organized in a network, with the seed gene-encoded proteins located in the center (Figure 6).

In line with the autophagy-annotated gene-related network in the cytoplasm, the up-regulated Gabarap was implicated in intracellular transport events associated with autophagosome maturation[22], [23], and Smpd1 was reportedly located in lysosomes and was found to play an important role in the metabolism of the lipid sphingomyelin[24]. Downregulation of mTOR and Klc1 may have an implication in the regulatory mechanisms of autophagy. mTOR was recognized as one of the most significant genes that may regulate autophagy since the negative regulation of mTOR by AMPK and p53 signaling may promote autophagy[25]. In addition, alternative splicing isoforms of Klc1 were reportedly involved in autophagy processes by binding cargos, such as vesicles, mitochondria, and the Golgi complex[26]. As for Pgrmc2, whether this molecule is critically involved in the regulation of autophagy warrants further research.

From these analysis, a clear connection can be seen between mTOR and autophagy, as mTOR has been reported as a negative regulator of autophagy[27], targeting at mTOR pathways may act as a new therapeutic direction.

### Increased autophagy activity and extended infiltration range of recurrent T cells

**Figure 7:**
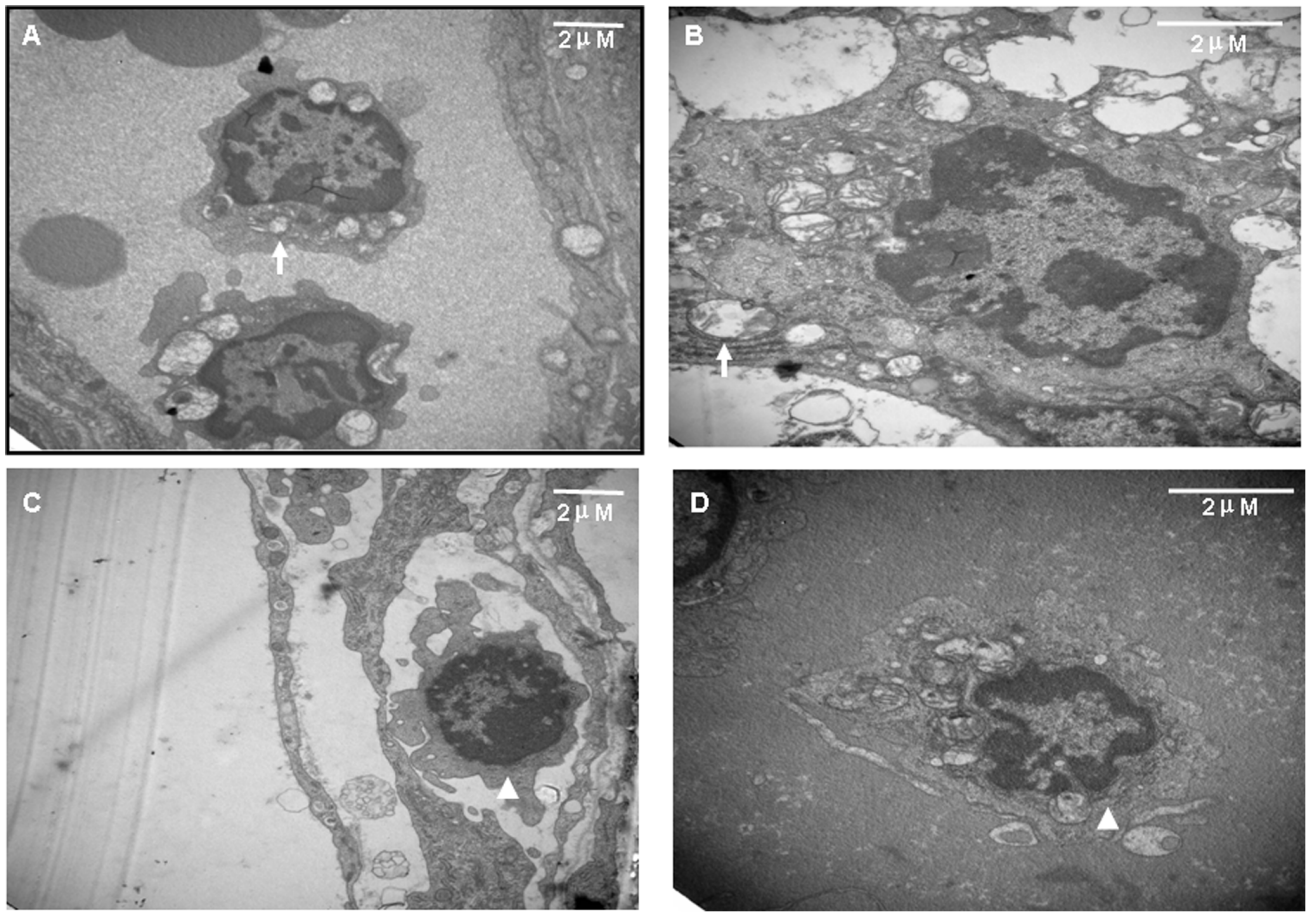
Increased autophagy activities in the recurrent uveitis T cells. Retinas were collected at the peak time point of monophasic uveitis and at the first peak time point of recurrent uveitis. Autophagosomes and autophagy vacuole (AV) numbers (Figure S1) were counted as an indicator of autophagy activity. In the recurrent EAU model, the infiltrated T cells in the blood–retinal barrier (A) and the local retina (B) showed an autophagy state; in the monophasic EAU model, the T cells in the blood–retinal barrier showed an inactivated state (C) and showed apoptosis or an inactivated state in the retina (D). Autophagosomes are indicated by arrows and apoptosis cells are indicated by arrowheads (scale bar: 2 µm).

**Figure 8:**
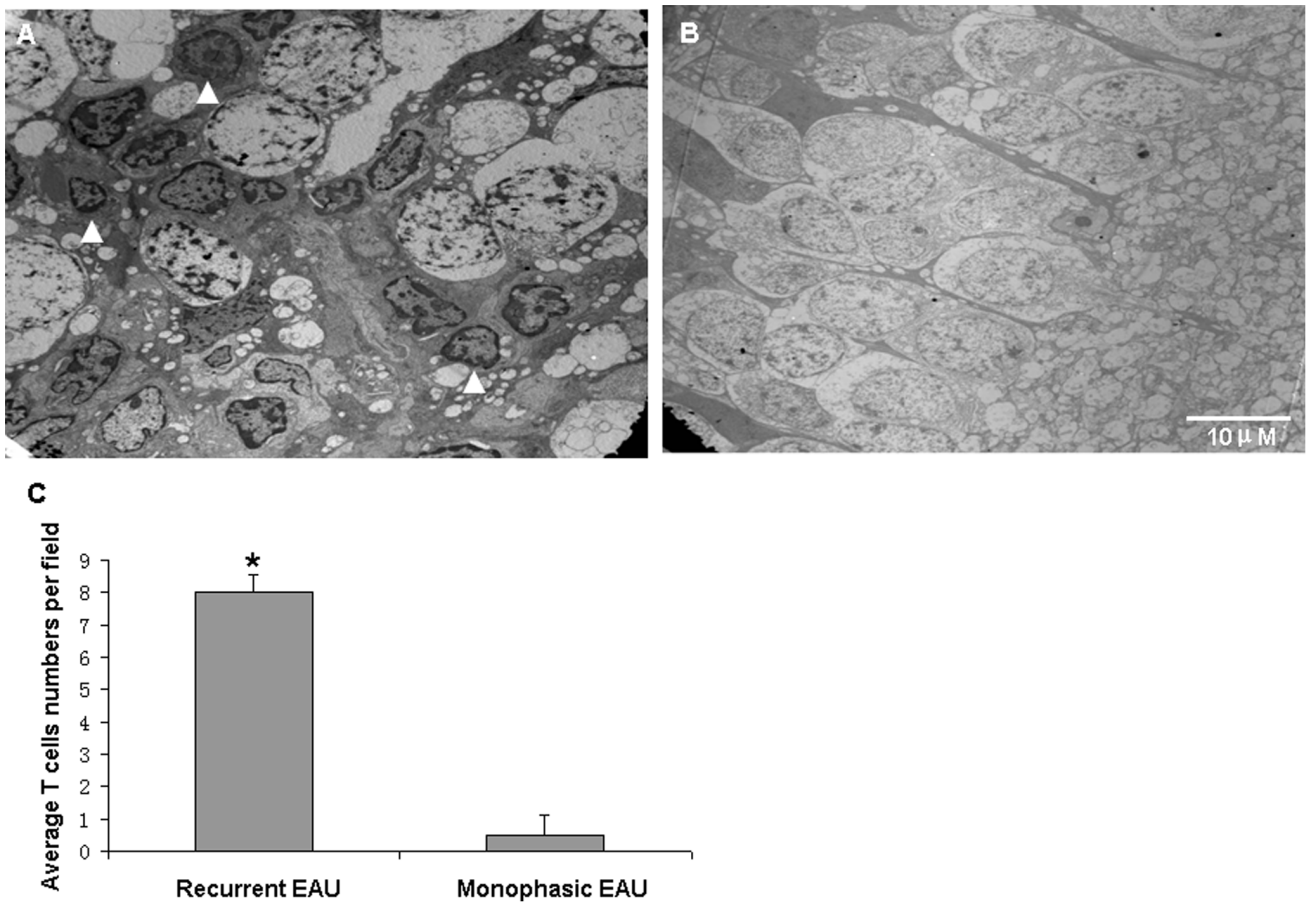
Distribution differences of the infiltrating T cells in recurrent and monophasic uveitis models. The recurrent uveitis T cells can be observed throughout the retina and reach to the retina ganglion cell layer (A, indicated by arrowheads); there were barely detectable monophasic uveitis T cells in the same area (B). The number of infiltrating T cells observed by TEM was quantified (C). Data represent three rats per group, three sections per rat; 20 fields/section (scale bar: 10 µm).

In order to testify whether autophagy activity was correlated with the recurrent disease process, we constructed recurrent and monophasic EAU models (Figure S1) and used TEM methods to detect the autophagy vacuoles (AV) in the infiltrated T cells at the blood–retinal barriers and retinas (Figure S2) at the disease peak time (14 days post immunization) of both models. The results showed that, the infiltrated T cells of the recurrent EAU at the blood–retinal barrier showed significant activation and increased autophagy activity with a notable number of AV in the cytoplasm as compared to the inactivated and apoptotic state of the T cells in the monophasic model (Figure 7). Furthermore, significantly more T cells with active autophagy activities in the recurrent model can reach to the ganglion cells layers of the retinas, whereas barely detectable T cells of the monophsic EAU can be found in the same area (Figure 8). On the other hand, the microglias in the monophasic model showed an activated state, whereas the phenomenon was not observed in the recurrent disease (Figure S3).

## Discussion

In the current study, by making use of publicly available databases, we proposed an integrative bioinformatics framework that linked gene expression profiles with interactome databases and protein location information to create a layered interactome. Grouped annotations were used to facilitate a synthetic description of the cellular biological processes involved in a particular disease phenotype. The analysis indicated that, as compared to the monophasic T cell line, the recurrent line showed significantly increased autophagy activity and mTOR may be a latent target to apply.

Furthermore, we established monophasic and recurrent uveitis models and used TEM methods to detect autophagy T cells *in vivo* to further support our results. Compared with the monophasic uveitis T cells, the recurrent uveitis T cells showed more autophagy activity in the blood–retina barrier and the retinas and reached to the ganglion cell layers of the retinas, indicating that autophagy had certain connections with disease recurrence, although the precise mechanisms need to be further investigated.

Previous work by Gerhild Wildner and co-workers, using the same data we analyzed in this assay, found that relapsing–remitting T cells appeared to have increased activation, survival, and enhanced Th1 cytokine (IFN-γ) production compared with monophasic T cells, which appeared to be more Th17 prone[3]. This results have also been confirmed by intracellular cytokine staining of intraocular cells from monophasic and relapsing uveitis, IFN-γ was the dominant cytokine in relapsing disease with a maximum expression during relapses; in contrast, intraocular T cells from monophasic disease showed increased populations coexpressing IFN-γ and IL-17 [28], [29].Combined with our data analysis, relapsing–remitting T cells showed more autophagy phenotype and more Th1 inclination because autophagy has been proven to promote the secretion of IFN-γ and maintain endoplasmic reticulum and mitochondrial homeostasis in activated T cells[15]. We therefore propose that autophagy may be the reason for relapsing–remitting T cells to be more Th1 prone. All these data are consistent with *in vivo* findings in the experimental autoimmune encephalomyelitis model in which MOG (Myelin Oligodendrocyte Glycoprotein)-specific Th1 responses were severely compromised in Beclin 1-deficient mice (Beclin 1 was required for the initiation of autophagasome formation), whereas Th17 responses were only modestly affected[16]. Moreover, low dose rapamycin exacerbated autoimmune experimental uveitis, and this action of rapamycin was thought to be mediated by autophagy[30]. These data suggest that autophagy is differentially required for the survival of T cell subsets, and different autophagy phenotypes may determine the disease phenotype, relapsing–remitting or monophasic.

While dysregulated or malfunctioned Tregs[31], [32], prolonged survival of autoreactive T cells or reactivation of long-life memory cells were demonstrated to contribute to recurrent autoimmune diseases, the mechanisms that underlie recurrent human uveitis remain unclear[3], [33], [34]. Our results indicated that the possible connection between autophagy and prolonged survival of autoreactive T cells or reactivation of long-life memory cells may be involved in the recurrent mechanisms. Our analysis has indicated that mTOR signaling, which is dependent on T cells to integrate immune signals and metabolic cues for their proper maintenance, activation, and differentiation[35], should become a priority when considering the potential therapeutic implications.

## Conclusions

The framework we proposed here, grouped annotations on the subcellular layered interactome, can decipher the most relevant biological process, which may point to a novel way to enhance the understanding of regulatory mechanisms behind a gene expression experiment. This report is the first to show that the recurrent EAU T cell line has an autophagy phenotype. Our results also indicate that targeting at mTOR pathways may perturb the pathology of recurrent autoimmune responses.

## Materials and Methods

### Fundamental issue with the data set used for the study and the specificity of the cell types

In order to decipher subtle differences, especially the difference in the initiation element in the specific effector phenotype of recurrent (R14) and monophasic uveitis (PDSAg) T cell lines, as Gerhild Wildner and co-workers intended to do in their previous microarray dataset analysis [3], the gene expression data of R14- and PDSAg-specific T cell lines were downloaded from the GEO (Gene Expression Omnibus) (accession number, GSE19652).

Briefly, so as to establish PDSAg- and R14 -specific T cell lines, Gerhild Wildner first set up active immunization by injection of peptide PDSAg derived from the sequence of bovine S-Ag (aa 341–354, FLGELTSSEVATEV), and peptide R14 from human IRBP (aa 1169–1191, PTARSVGAADGSSWEGVGVVPDV), to induce monophasic and recurrent uveitis in Lewis rats. In brief, Lewis rats were immunized subcutaneously into both hind legs with a total volume of 200 µl emulsion containing 15 µg peptide R14 or PDSAg, both in complete Freund's adjuvant and fortified with Mycobacterium tuberculosis strain H37RA to a final concentration of 2.5 mg/ml. Uveitis was graded clinically and the clinical course were shown in [36].

Then, lymph nodes were collected 10 days post active immunization and PDSAg- and R14 -specific T cell lines were generated by alternating cycles of antigen stimulation in the presence of Antigen Presenting Cells (APC, thymocytes of naïve Lewis rats that irradiated with 20 Gy). After restimulation, the APC were removed from the cell cultures using a Ficoll density gradient centrifugation and the remaining T cells were stored at −80°C until RNA preparation. In order to obtain antigen specific T cell lines, 2–4 re-stimulations of the cultured lymphocytes were performed. The antigen specificity of T cell lines was then tested by proliferation assays measured by ^3^H-thymidine incorporation. These procedures ensure that the established cell lines are the auto-antigen specific T cells. PDSAg-specific T cell lines were testified to induce monophasic uveitis disease process, whereas R14-specific T cell lines were proved to induce recurrent uveitis process through adoptively transfer, yet the R14-mediated relapses are generally less severe and unpredictable with respect to clinical onset and intensity [3].

### Layered interactomes

The differentially expressed genes between relapsing–remitting and monophasic T cell lines were identified using Student's *t* test filter (p<0.05). The differential genes were further clustered by using the CLICK algorithm[37], which utilized graph-theoretic and statistical techniques to identify tight groups of highly associated clusters in order to detect genes with similar expression patterns, i.e., up-regulated or down-regulated genes.

There are few protein interaction databases that are available for *Rattus norvegicus*. Therefore, we used the FunCoup database to set up the initial interactome of the supposed proteins encoded by the differentially expressed genes. FunCoup is a statistical framework of data integration for identifying functional coupling between proteins. It transfers information from model organisms via orthologs found using the In Paranoid program[38]. A traditional interactome was assembled by merging the FunCoup database with proteins encoded by the differentially expressed genes that were obtained from the dataset. Single nodes, small components of the interactome, duplicated edges, and self-loops were removed prior to the analysis. Furthermore, information on the protein subcellular localization was retrieved from the HPRD database[8]. Cerebral[10], a Cytoscape plugin, was applied to redistribute the nodes and the interactome into different layers. Nodes without localization information were placed in the same layer as that of their closest neighbors.

### Annotations grouping analysis

To determine the underlying events in the different layers of the interactomes, differential genes in the different layers were annotated in the context of the GO and KEGG databases, using the DAVID functional annotation tool; the Bonferroni *P* test was applied to identify significantly over-expressed terms (*p*<0.05). To avoid being overwhelmed by a long list of terms, ClueGO, another Cytoscape plugin, was used to combine the highly associated terms to reduce redundancy and to reflect the relationships among these selected terms based on the similarity of their related genes. In brief, a binary gene-term matrix with the selected terms and their associated genes was created; then based on this matrix, a term–term similarity matrix was calculated using chance-corrected kappa statistics to determine the association strength between the terms. Grouped annotations were created by iterative merging of initially defined groups based on the predefined kappa score threshold (>0.3)[11]. The most significantly up-regulated and/or down-regulated annotated terms for each group were chosen as the labels of the groups. In order to provide more detailed and representative information about the grouped annotations in addition to the most significantly regulated terms, the most relevant annotation terms are also listed (in italics) in groups 4–6. The proportion of up-regulated or down-regulated genes in each group determined the direction of either enhanced or decreased reactions, i.e., up-regulated and/or down-regulated grouped annotations.

### Induction and clinical evaluation of monophasic and recurrent experimental autoimmune uveitis

Female Lewis rats (160–180 g, 5–6 weeks) of specified pathogen-free grade were purchased from Peking Vital River Laboratory Animal Ltd. (Beijing, China). All rats were fed and maintained in specified pathogen-free conditions and processed according to the guidelines of Care and Use of Laboratory Animals published by the China National Institute of Health. The rats were injected with a combination of intramuscular ketamine hydrochloride 50 mg/kg and xylazine hydrochloride 5 mg/kg to induce anesthesia and analgesia. All experimental procedures adhered to the Association for Research in Vision and Ophthalmology Statement for the use of animals in ophthalmic and vision research. The study was approved by the Animal Ethics Committee of Harbin Medical University, China (Permit Number: HMUIRB20140016).

Peptides from human IRBP-R16 (residues 1177–1191, ADGSSWEGVGVVPDV) and R14 (residues 1169–1191, PTARSVGAADGSSWEGVGVVPDV) were used to induce monophasic and recurrent uveitis. Three batches of immunization, each including 14 Lewis rats (randomly selected rats for monophasic [n = 7] and recurrent [n = 7] uveitis induction) were used. The immune process followed that of our previously published paper [1]; briefly, 50 µg peptide emulsification with Freund's complete adjuvant (Sigma-Aldrich, St. Louis, MO) was injected in the rat footpads. Concurrently, both groups of animals were intraperitoneally injected with 1 µg of pertussis toxin (Sigma-Aldrich).

Animals were monitored daily. Clinical signs were scored by slit-lamp biomicroscopy according to the following criteria[1]: 0, the eye is translucent and reflects light; 0.5, dilated blood vessels in the iris; 1.0, engorged blood vessels in the iris, abnormal pupil contraction; 2.0, hazy anterior chamber, decreased red reflex; 3.0, moderately opaque anterior chamber but pupil still visible, dull red reflex; 4.0, opaque anterior chamber and obscured pupil, red reflex absent, proptosis.

### Transmission electron microscopy

The entire eye balls of euthanized animals at the peak period at 14 days post immunization (dpi) with both of the disease models (three rats per group) were freshly dissected and fixed in 1% freshly made paraformaldehyde with 2% glutaraldehyde for 24 h before the corneas were cut and lens were carefully removed. Samples were fixed for 2 h in 1% osmium tetroxide and dehydrated in graded ethanol and embedded in araldite. Semi-thin sections of the eyes were cut to locate the optic nerve parts of the block, as the inflammation in these layers was the most serious. Then, three ultra-thin sections per rat were cut and stained with uranylacetate and lead citrate and then observed with TEM for 20 fields/section (Hitachi, H-765; Japan).

## Supporting Information

Figure S1 **Time course of clinical disease from rats immunized with R-16-CFA (A) and rats after immunization with R-14-CFA (B).** The curves show the inflammation of single eyes; grey symbols, right eyes and black symbols, left eyes. Each diagram represents one individual animal.
(TIF)

Figure S2 **Increased autophagy activity in recurrent uveitis T cells compared with monophasic uveitis T cells.** TEM showed more autophagy vacuoles (AV) in infiltrating T cells in recurrent uveitis compared with monophasic uveitis. The T cell autophagy observed by TEM was quantified by either percentage of AV-positive T cells (A) or the total number of AV (B). Data represent three rats per group, three sections per rat; 20 fields/section (*p*<0.05).
(TIF)

Figure S3 **The activated microglias in plexiform layers of the retina in monophasic uveitis.** TEM showed that no infiltrated or activated microglias could be observed in the plexiform layers of recurrent uveitis (A), while activated microglias could be observed in the plexiform layers of monophasic uveitis (B) (indicated by arrows). The number of infiltrating microglias observed by TEM was quantified (C). Data represent three rats per group, three sections per rat; 20 fields/section (scale bar: 10 µm).
(TIF)

File S1 **Supporting tables. Table S1,** Annotation grouped in plasma membrane layers. The Cytoscape and Cerebral plugin were used to establish the subcellular layered interactome based on differential expression, global interactome, and subcellular localization information. Then, the layered interactomes were grouping annotated by the ClueGO plugin based on Gene Ontology and Kyoto Encyclopedia of Genes and Genomes databases. Such grouped annotations in the plasma membrane layers were listed to recognize the relationships among these processes. **Table S2,** Annotation grouped in cytoplasm layers. The Cytoscape and Cerebral plugin were used to establish the subcellular layered interactome based on differential expression, global interactome, and subcellular localization information. Then, the layered interactomes were grouping annotated by the ClueGO plugin based on Gene Ontology and Kyoto Encyclopedia of Genes and Genomes databases. Such grouped annotations in the cytoplasm layers were listed to recognize the relationships among these processes. **Table S3,** Annotation grouped in nucleus layers. The Cytoscape and Cerebral plugin were used to establish the subcellular layered interactome based on differential expression, global interactome, and subcellular localization information. Then, the layered interactomes were grouping annotated by the ClueGO plugin based on Gene Ontology and Kyoto Encyclopedia of Genes and Genomes databases. Such grouped annotations in the nucleus layers were listed to recognize the relationships among these processes.
(XLS)
