# Correction: Grouping Annotations on the Subcellular Layered Interactome Demonstrates Enhanced Autophagy Activity in a Recurrent Experimental Autoimmune Uveitis T Cell Line

**DOI:** 10.1371/journal.pone.0126597

**Published:** 2015-04-16

**Authors:** Xiuzhi Jia, Jingbo Li, Dejing Shi, Yu Zhao, Yucui Dong, Huanyu Ju, Jinfeng Yang, Jianhua Sun, Xia Li, Huan Ren

## Notice of Republication

This article was republished on March 3, 2015, to remove confidential or copyrighted material and to correct a name in reference 3.

In addition, a reader recently pointed out some overlap between the text in the article and that from some previous publications. This text should not have been used verbatim. The authors would like to thank the reader and apologize for the overlap in text.

In the first paragraph of the Introduction and in the Methods, there are sentences that overlap with text in reference 3, and in the third paragraph of the Discussion, there is a sentence that overlaps with text in reference 29.

Even though we cited the articles above, we apologize that the information was not rewritten more carefully. The issue of existing text overlap has no bearing on the results and conclusions of the study.
