# Supplementary figures and images for: Grouping Annotations on the Subcellular Layered Interactome Demonstrates Enhanced Autophagy Activity in a Recurrent Experimental Autoimmune Uveitis T Cell Line
Source: PLoS One. 2014 Aug 12;9(8):e104404. doi: 10.1371/journal.pone.0104404 (PMC4130584; doi:10.1371/journal.pone.0104404)

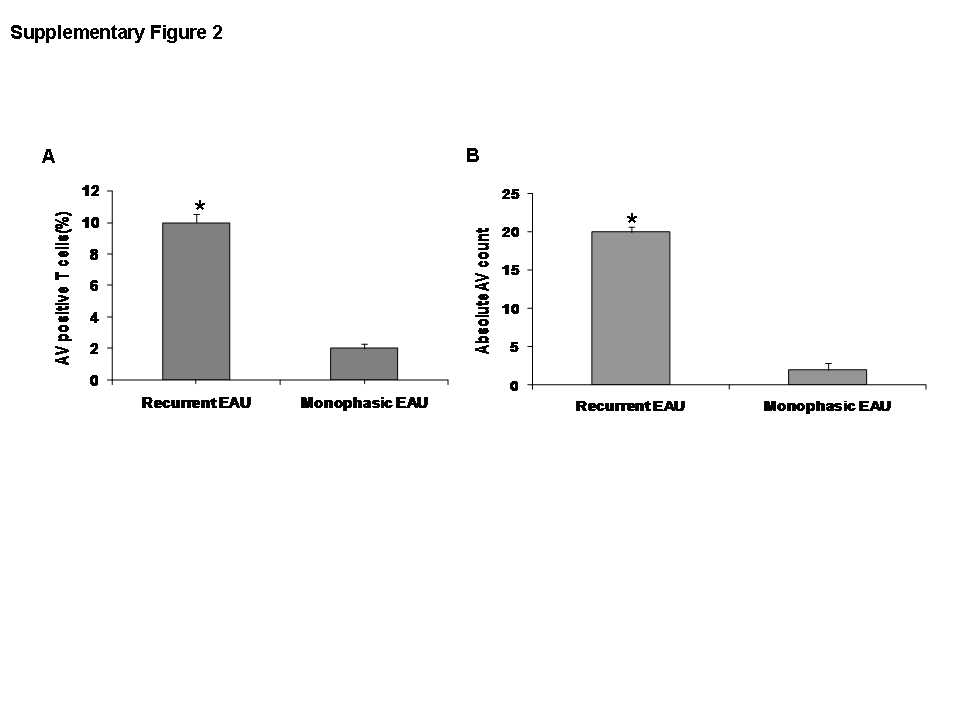

Supplement: Figure S2 — Increased autophagy activity in recurrent uveitis T cells compared with monophasic uveitis T cells. TEM showed more autophagy vacuoles (AV) in infiltrating T cells in recurrent uveitis compared with monophasic uveitis. The T cell autophagy observed by TEM was quantified by either percentage of AV-positive T cells (A) or the total number of AV (B). Data represent three rats per group, three sections per rat; 20 fields/section (p<0.05). (TIF) [file pone.0104404.s002.tif]
